# Impressions of Modeling Compound

**Published:** 1897-06

**Authors:** 


### Editorial, Etc.

### IMPRESSIONS OF MODELING COMPOUND.

Dr. W. E. Robertson, of Alvarado, Texas, suggests the following method for perfecting impressions of modeling compound :

Secure the impression in the ordinary manner, and after removal from the mouth, and when it has become quite hard, trim off any surplus until it corresponds to the shape of the rubber plate or base to be constructed.

Then hold the inner surface of the modified impression over the flame of a spirit lamp, or Bunsen burner, until the surface of the impression only is softened, but not the main body of the impression. Then return the impression while it is warm to the mouth, and after firmly pressing it home, allow it to harden before removing it.

Dr. Robertson claims that such a method will give more satisfactory results than the ordinary manipulation of modeling compound.

